# High-performance analog signal processing with photonic integrated circuits

**DOI:** 10.1038/s41377-025-01806-0

**Published:** 2025-03-27

**Authors:** Francesco Morichetti

**Affiliations:** Dipartimento di Elettronica, Informazione e Bioingegneria, Politecnico di Milano, Milano, Italy

**Keywords:** Microwave photonics, Integrated optics

## Abstract

Digital processing is our preferred way to manipulate data, as it gives us unparalleled flexibility. However, as the volume of information increases, fully digital electronic solutions are encountering memory, latency, and power challenges. A renewed interest is growing in analog signal processing, and photonics integrated circuits could really be a game-changing technology.

## Graphical abstract

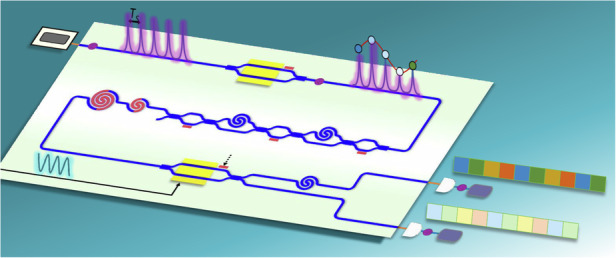

The rapid expansion of cloud-based services, high-speed mobile networks, Internet of Things (IoT) applications, and the emergence of unmanned vehicles are placing an increasing burden on existing digital electronic hardware that implements data processing systems^1^. Traditional digital signal processing relies on analog-to-digital converters (ADCs) and large-capacity memory, which become increasingly demanding as data volume grows. This pressure is further enhanced by the evolution of artificial intelligence (AI) and high-performance computing systems, which will require soon hardware platforms with speed, latency, and energy consumption performance well beyond those offered by traditional electronic circuits^2^. It seems we are reaching a turning point: although fully digital solutions offer the highest flexibility and reconfigurability, alternative hardware technologies are becoming necessary for a sustainable growth of next-generation AI-driven systems.

Promising solutions may be offered by hybrid schemes, where some signal processing tasks are conveniently handled in the analog domain^3^. Recent works have shown indeed how analog electronic circuits provide an efficient alternative to digital ones to minimize the time and energy spent for computations^4^. Interestingly, some analog functions can be efficiently performed in the optical domain.

Microwave photonics (MWP)^5^, that is the manipulation of high-frequency signals with optical technologies, has been known for several decades, but only now MPW is being considered seriously as a viable route (or maybe the only viable route) to support high-speed digital signal processing. Compared to pure electronic systems, upconverting radiofrequency signals into the optical domain offers significantly broader bandwidth, and the lack of analog-to-digital conversion enables extremely low latency and power consumption^6,7^, Further, MWP schemes can be efficiently implemented on small-form chip-scale systems^8^ and can benefit from the maturity level of current photonic integrated platforms^9^. To give an example, in their work published in *Light: Science & Applications*, *Weipeng Zhang and coworkers* from Princeton University, NJ, and Queen’s University, Canada, developed a silicon photonic integrated processor to solve dynamic interference of RF signals in real-time through blind source separation (BSS). Processing the signals in the analog optical domain enables extremely rapid de-mixing and recovery of the signal-of-interest in about ten picoseconds, reducing the latency of electronic counterparts by more than three orders of magnitude.

Most integrated MPW subsystems developed so far have been implemented as application-specific photonic integrated circuits (ASPICs), which are custom-designed chips that perform specific functions, but lack reconfigurability and are not suitable for multifunctional applications. Evolution towards more flexible or even general-purpose integrated MPW architectures would allow the penetration of analog photonic processing in many fields of application. Recently some examples of general-purpose analog photonic processors have been proposed^10^, which are based on programmable photonic integrated circuits^11^ and enable the implementation of different functions through circuit reconfiguration.

A significant step forward in the field of analog optical processing has been recently published in *Light: Science & Applications* by Na Qian and coworkers from *Shanghai Jiao Tong University, Peking University, and Zhejiang University, China*. In this work, the Authors demonstrated a multifunctional analog parallel processor (APP) for broadband integrated systems, which is integrated in a small silicon photonic chip of only 12.6 mm^2^ (see Fig. 1)^12^. The proposed APP discretizes and parallelizes broadband signals directly in the optical domain, thus reducing significantly the computational complexity and eliminating the need for high-speed memory that is traditionally required for serial-to-parallel conversion. To this end, optical frequency combs are utilized for temporal sampling of the broadband analog signal. After the sampling, the discretized signals are parallelized through fast integrated switches, which are implemented with high-speed Mach-Zehnder modulators (MZMs), and are routed into 2 ^*N*^ parallel sub-sequences. As a consequence, in each sub-channel, the effective data rate is reduced by a factor 2 ^*N*^ in such a way that the clock rate and the computing resources of the subsequent computing units are mitigated. To finely handle signal synchronization, tuneable time delay lines (TDLs) are also integrated onto the same photonic chip. Notably, the system does not include high-speed memories for serial-parallel conversion, which are instead necessary in traditional electrical digital processors.

**Fig. 1:**
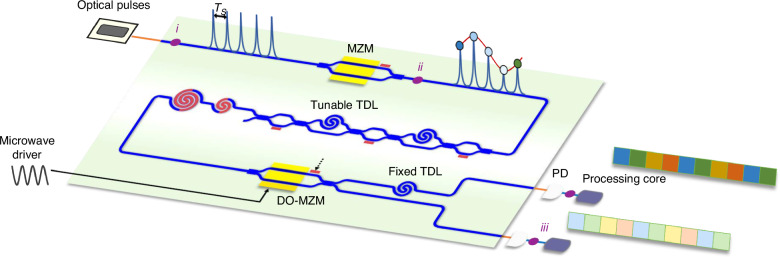
Schematic of the silicon photonic chip hosting the analog parallel processor (APP). The optical pulses generated by a comb source perform temporal discretization of the broadband RF signal driving the Mach-Zehnder modulator (MZM). A tunable time delay line (TDL) synchronizes the discretized signal with the microwave driver of the dual-output modulator (DO-MZM) used as a switch to parallelize the temporal discrete signal into the two output channels^12^

With respect to related research, the circuit implemented in ref.^12^ is not application specific and indeed its functionality is validated in two different applications, that are the processing of 6 GHz radar signals and the downsampling of 16 QAM communication signals at a data rate of 8 Gbit/s. In the reported experiments the photonic chip hosts only a two-channel processing unit, but current integrated photonic technologies would enable the implementation of architectures with a higher number of parallel channels. The main limit to parallelization is posed by the reduction of optical power per channel, which is responsible for a degradation of the signal-to-noise ratio. Future implementations of large parallel systems on chip would benefit from the integration of optical amplifiers, as well as pulse generators and high-speed photodetectors. To this end, hybrid silicon photonic platforms integrating III-V active devices, such as lasers and amplifiers, could offer all the required functions with a density of integration^9^.

Beyond ranging and communication applications, analog photonic processors are expected to play an increasingly significant role in supporting electronic digital signal processing across various fields. Recent studies have demonstrated the capability of photonic processors to perform mathematical operations with significantly lower latency and energy consumption compared to digital electronics. Notable examples include photonic matrix-vector multiplication and its applications in photonic neural networks^13,14^, matrix factorization and singular value decomposition^15^; equation solving and matrix inversion^16^; arbitrary beam-front generators^17^, analyzers^18^, and distortion compensators^19^. Additionally, cognitive sensing^20^ and optical semantic communication^21^ are emerging areas where analog optical processing is envisioned as a key enabling technology.

While analog signal processing is unlikely to completely replace digital processing—just as photonic processors will not replace entirely electronic ones - growing evidence suggests that future systems will benefit from hybrid hardware platforms that integrate analog and digital electronics with analog photonic processors. Recently, the first examples of all-analog chips, leveraging the advantages of both analog electronics and analog photonics, have been proposed for high-speed vision tasks, demonstrating performance improvements by three orders of magnitude compared to state-of-the-art computing processors (with more than 99% operations implemented by optics)^22^. The role and the impact of analog photonic processing are getting more and more evident and its penetration is expected both in high-performance computing systems and in edge computing devices.
